# Detection of livestock-associated MRSA ST398/t011 and hospital-associated MRSA ST239/t037 in a referral hospital in northeastern Iran: A six-month cross-sectional surveillance study (July-December 2025)

**DOI:** 10.1016/j.nmni.2026.101834

**Published:** 2026-08-27

**Authors:** Seyed Ahmad Hashemi, Ali Haghbin, Ahmad Vosughi Motlagh, Amir Azimian

**Affiliations:** a Vector-borne Diseases Research Center, North Khorasan University of Medical Sciences, Bojnurd, Iran; b Department of Pediatrics, Faculty of Medicine, North Khorasan University of Medical Sciences, Bojnurd, Iran; c Department of Pathobiology and Laboratory Sciences, Faculty of Medicine, North Khorasan University of Medical Sciences, Bojnurd, Iran

**Keywords:** Methicillin-resistant *Staphylococcus aureus*, MRSA, Livestock-associated, Iran, Molecular epidemiology, Antimicrobial resistance

## Abstract

**Background:**

Following initial detection of livestock-associated MRSA (LA-MRSA) ST398/t011 and hospital-associated MRSA (HA-MRSA) ST239/t037 in early 2025, we conducted a cross-sectional six-month surveillance study to determine whether these clones represent transient introductions or continued detection in a referral hospital in Bojnurd, North Khorasan Province, northeastern Iran.

**Methods:**

From July to December 2025, we collected 258 consecutive clinical specimens at Imam Reza Hospital, Bojnurd. MRSA isolates underwent antimicrobial susceptibility testing, *SCCmec* typing, *spa* typing, and multilocus sequence typing. PCR detected genes encoding Panton-Valentine leukocidin (PVL), toxic shock syndrome toxin (TSST-1), and other virulence factors. Livestock exposure was defined using a structured questionnaire.

**Results:**

Among 61 *S. aureus* isolates (23.6%), 11 (18.0%) were MRSA, all belonging exclusively to two previously identified clones: ST398/t011/*SCCmec* V/*agr* I (n = 6; 54.5%) and ST239/t037/*SCCmec* III/*agr* I (n = 5; 45.5%). Resistance patterns remained clone-specific, with all ST398 isolates resistant to tetracycline and trimethoprim/sulfamethoxazole. The PVL gene was detected in one ST398 isolate (16.7%). All ST239 isolates carried *tst* and *sec*. Livestock exposure was reported in 83.3% of ST398-infected patients. Clinically, all 11 patients survived; only one required ICU admission, and none experienced treatment failure.

**Conclusion:**

This study documents continued detection of LA-MRSA ST398/t011 at a referral hospital in Bojnurd, co-circulating with endemic HA-MRSA ST239/t037. The detection of a PVL-positive ST398 isolate warrants further investigation and underscores the importance of integrated One Health surveillance. Given the small sample size (n = 11), these findings are descriptive and hypothesis-generating rather than confirmatory.

## 1 Introduction

Methicillin-resistant *Staphylococcus aureus* (MRSA) remains a leading cause of healthcare-associated and community-onset infections globally, with substantial morbidity, mortality, and healthcare costs [1,2]. The molecular epidemiology of MRSA is defined by the emergence and dissemination of distinct clonal lineages, each with specific ecological niches, resistance profiles, and virulence gene repertoires [3,4].

Traditionally, MRSA clones have been classified as healthcare-associated (HA-MRSA) or community-associated (CA-MRSA) based on genetic and epidemiological characteristics [5]. Over the past two decades, a third category has emerged: livestock-associated MRSA (LA-MRSA), primarily affecting individuals with direct occupational exposure to production animals, particularly swine, cattle, and poultry [6,7].

The most widely disseminated LA-MRSA clone is sequence type 398 (ST398), typically carrying *SCCmec* type IV or V and belonging to *spa* types t011, t034, or t108 [8,9]. First identified in 2003 in France and subsequently reported across Europe, North America, Asia, and Australia, ST398 has become a paradigm of zoonotic bacterial adaptation [10,11]. Initially considered a low-virulence colonizer of animals, ST398 is now increasingly recognized as a cause of severe human infections, including skin and soft tissue infections, necrotizing pneumonia, osteomyelitis, and bloodstream infections [12,13].

Of particular concern is the acquisition of Panton-Valentine leukocidin (*PVL*) by LA-MRSA ST398. *PVL* is a bicomponent pore-forming toxin associated with severe necrotizing infections and was historically rare in classical LA-MRSA strains [14,15]. Recent reports from Europe, Asia, and Australia have documented the emergence of *PVL*-positive ST398, suggesting horizontal acquisition of *PVL*-encoding prophages and potential adaptation to human hosts [16,17].

In Iran, MRSA is endemic in most tertiary care hospitals, with HA-MRSA ST239/*spa* t037/SCC*mec* III constituting the predominant healthcare-associated lineage for nearly two decades [18,19]. This pandemic clone, also known as the Brazilian/Hungarian clone, is characterized by multidrug resistance, including resistance to macrolides, aminoglycosides, fluoroquinolones, and increasingly, mupirocin and rifampicin [20,21]. Despite extensive livestock farming and close human-animal contact in many regions of Iran, LA-MRSA ST398 has rarely been reported [22].

In our recent publication in this journal, we reported the first documented clinical detection of LA-MRSA ST398/t011 in Iran, co-circulating with HA-MRSA ST239/t037 in patients at Imam Reza Hospital, Bojnurd, during the first half of 2025 [23]. This unexpected finding raised critical questions: Was this a sporadic, self-limited event, or did it signal continued presence of a novel MRSA lineage in this setting?

To address this question, we conducted a prospective six-month active surveillance study from July to December 2025. Our objectives were to: (i) determine the prevalence and clonal distribution of MRSA in the second half of 2025; (ii) characterize the phenotypic and genotypic resistance profiles of recovered isolates; (iii) assess virulence gene carriage, particularly PVL; and (iv) compare findings with our first-half data to evaluate continued detection.

## 2 Materials and methods

### 2.1 Study design and setting

This prospective cross-sectional surveillance study was conducted from July 1 to December 31, 2025, at Imam Reza Hospital, Bojnurd, the primary tertiary care referral center for North Khorasan province, northeastern Iran. This study is a direct continuation of our previously published first-half 2025 surveillance (January-June 2025) [23], conducted at the same hospital using identical inclusion criteria, laboratory methods, and analysis protocols. North Khorasan is an agriculturally intensive region with extensive livestock farming, including cattle feedlots, sheep husbandry, and poultry production, and high rates of human-animal contact in rural and peri-urban communities. The hospital has 350 beds and serves a catchment population of approximately one million.

### 2.2 Sample collection and bacterial isolation

Consecutive non-duplicate clinical specimens were collected from patients with clinically suspected infections, defined as: (i) fever (temperature ≥38°C) or hypothermia (<36°C); (ii) local signs of infection (erythema, swelling, warmth, purulent discharge); and (iii) appropriate systemic symptoms. Specimens were transported immediately to the hospital microbiology laboratory. Specimen types included: wound swabs/pus, blood, respiratory secretions (sputum, tracheal aspirate, bronchoalveolar lavage), urine, and other sterile body fluids. Only one isolate per patient was included.

Specimens were cultured on blood agar and mannitol salt agar (Merck, Germany) and incubated aerobically at 37°C for 24-48 h. *S. aureus* was identified based on: (i) colony morphology (golden-yellow, beta-hemolytic colonies); (ii) Gram stain (Gram-positive cocci in clusters); (iii) positive catalase reaction; (iv) positive tube coagulase test; and (v) mannitol fermentation.

### 2.3 Antimicrobial susceptibility testing

Antimicrobial susceptibility was determined using the Kirby-Bauer disk diffusion method on Mueller-Hinton agar (Merck, Germany) according to CLSI 2025 guidelines (M100-ED34) [24]. The following antibiotic disks (Mast Diagnostics, UK) were tested: Cefoxitin (30 μg), Penicillin G (10 U), Tetracycline (30 μg), Erythromycin (15 μg), Clindamycin (2 μg), Levofloxacin (5 μg), Gentamicin (10 μg), Trimethoprim/sulfamethoxazole (1.25/23.75 μg), Mupirocin (200 μg), Rifampicin (5 μg), Linezolid (30 μg), Vancomycin (30 μg). Vancomycin MIC was determined using E-test strips (bioMérieux, France). D-test was performed to detect inducible clindamycin resistance by placing erythromycin (15 μg) and clindamycin (2 μg) disks 15-20 mm apart; flattening of the clindamycin zone adjacent to erythromycin (D-shaped zone) was interpreted as positive. *S. aureus* ATCC 25923 and ATCC 29213 served as quality controls.

### 2.4 Definition of livestock exposure

Livestock exposure was assessed using a structured questionnaire administered to all patients or their next-of-kin. Exposure was defined as self-reported direct contact with livestock (cattle, sheep, goats, or poultry) within 12 months preceding specimen collection, including: (i) occupational exposure (farming, ranching, veterinary care, or slaughterhouse work); (ii) household exposure (living on a farm with livestock or regular animal contact); or (iii) rural/agricultural residence with documented animal contact. Exposure frequency was categorized as daily, weekly, or occasional. Information on family members' livestock exposure was also collected.

### 2.5 DNA extraction

Genomic DNA was extracted from overnight cultures using the boiling lysis method. Briefly, 3-5 colonies were suspended in 200 μl of Tris-EDTA buffer (10 mM Tris-HCl, 1 mM EDTA, pH 8.0), heated at 95°C for 15 min, cooled on ice for 5 min, and centrifuged at 12,000 rpm for 5 min. The supernatant containing DNA was transferred to sterile tubes and stored at −20°C. DNA concentration and purity were assessed using a Nanodrop ND-1000 spectrophotometer (Thermo Fisher Scientific, USA).

### 2.6 Confirmation of MRSA and detection of *mecA*

All cefoxitin-resistant isolates (zone diameter ≤21 mm) were subjected to PCR amplification of the *mecA* gene using previously described primers [25]. PCR conditions included: initial denaturation at 94°C for 5 min; 30 cycles of denaturation at 94°C for 30 s, annealing at 55°C for 30 s, extension at 72°C for 1 min; and final extension at 72°C for 7 min. PCR products were electrophoresed on 1.5% agarose gels stained with DNA Safe stain (Sinaclon, Iran) and visualized under UV transillumination.

### 2.7 *SCCmec* typing

SCC*mec* typing was performed using multiplex PCR strategies described by Boye et al. [26] and Milheiriço et al. [27], enabling differentiation of SCC*mec* types I through V based on amplification of specific *ccr* and *mec* gene complexes.

### 2.8 *agr* typing

Accessory gene regulator (*agr*) specificity groups (I-IV) were determined using a multiplex PCR assay targeting the hypervariable region of the *agr* locus, as described by Gilot et al. [28].

### 2.9 *spa* typing

Amplification of the polymorphic X region of the protein A gene (*spa*) was performed using primers *spa*-1113f (5′-TAAAGACGATCCTTCGGTGAGC-3′) and *spa*-1514r (5′-CAGCAGTAGTGCCGTTTGCTT-3′) [29]. PCR products were sequenced bidirectionally using Sanger sequencing on an ABI 3730xl DNA Analyzer (Applied Biosystems, USA) at Macrogen Inc., South Korea. Sequencing data were analyzed using Ridom StaphType software version 2.2.1 (Ridom GmbH, Germany) to assign *spa* types.

### 2.10 Multilocus sequence typing (MLST)

MLST was performed according to Enright et al. [30]. Seven housekeeping genes were amplified by PCR and Sanger sequenced. Allelic profiles and sequence types (STs) were assigned using the *S. aureus* MLST database (https://pubmlst.org/saureus/).

### 2.11 Detection of virulence genes

PCR was used to screen for the presence of four virulence-associated genes: Panton-Valentine leukocidin (*lukS/F-PV*), toxic shock syndrome toxin (*tst*), enterotoxin C (*sec*), and alpha-hemolysin (*hla*), as described previously [31,32].

### 2.12 Statistical analysis

Data were entered into SPSS version 26.0 (IBM Corp., Armonk, NY, USA). Descriptive statistics (frequencies, percentages, means, ranges) summarized demographic, clinical, and microbiological characteristics. Categorical variables were compared between clone groups using Fisher's exact test. For the comparison of livestock exposure between clone groups, 95% confidence intervals for proportion differences were calculated. A p-value <0.05 was considered statistically significant. Given the small sample size and multiple comparisons, results should be interpreted as descriptive and hypothesis-generating. No correction for multiplicity was applied.

### 2.13 Ethical considerations

This study was approved by the Ethics Committee of North Khorasan University of Medical Sciences (Approval No**.** IR.NKUMS.REC.1401.012). Patient data were anonymized, and informed consent was waived due to the observational, non-interventional nature of the study.

## 3 Results

### 3.1 Study population and MRSA prevalence

During the six-month surveillance period, 258 non-duplicate clinical specimens were collected. Specimen distribution was: wound/pus (98/38%), blood (62/24%), respiratory (52/20.2%), urine (38/14.7%), and other sterile sites (8/3.1%). Of these, 61 (23.6%) were culture-positive for *S. aureus*. MRSA was confirmed in 11 isolates, representing 18.0% of *S. aureus* isolates and 4.3% of total specimens.

### 3.2 Demographic and clinical characteristics of MRSA-positive patients

The 11 MRSA-positive patients comprised 6 males (54.5%) and 5 females (45.5%), age range 8 to 60 years (median: 33 years; mean: 33.8 ± 16.0 years). MRSA isolates were distributed by specimen type: wound (6/54.5%), blood (3/27.3%), respiratory (1/9.1%), urine (1/9.1%), and other sterile sites (0%).

**Clinical outcomes:** length of stay ranged from 5 to 28 days (mean 12.5 ± 6.8 days); ICU admission was required for 1 patient (9.1%); surgical intervention for 3 patients (27.3%); treatment failure (persistent infection requiring antibiotic change or hospitalization >14 days) occurred in 0 patients; and 30-day mortality was 0/11 (0%).

Livestock exposure was systematically queried for all MRSA-positive patients. Among the 5 ST398 patients with livestock exposure: 2 had occupational exposure (veterinarian, farmer), 2 had household/family contact, and 1 had rural/agricultural residence. Additionally, for 5/6 ST398 patients, at least one family member also had documented livestock exposure. No ST239/t037 patient (n = 5) reported livestock contact (p = 0.015, Fisher's exact test**;** 95% CI for proportion difference: 29.4% to 100.0%). Detailed characteristics are presented in Table 1.

**Table 1:** Demographic and clinical characteristics of 11 MRSA-Positive patients.

Patient ID	Age (years)	Sex	Clone (ST/spa)	Specimen Type	Livestock Exposure	Length of Stay (days)	ICU Admission	Surgery Required	Treatment Failure	30-day Mortality
1	42	M	ST398/t011	Wound	Yes (occupational)	10	No	Yes	No	No
2	28	F	ST398/t011	Blood	Yes (household)	8	No	No	No	No
3	45	M	ST398/t011	Wound	Yes (occupational)	7	No	No	No	No
4	60	M	ST398/t011	Respiratory	Yes (rural)	14	No	No	No	No
5	33	F	ST398/t011	Urine	Yes (household)	6	No	No	No	No
6	22	F	ST398/t011	Blood	No	5	No	No	No	No
7	18	M	ST239/t037	Wound	No	28	Yes (3 days)	No	No	No
8	55	M	ST239/t037	Blood	No	15	No	Yes	No	No
9	38	F	ST239/t037	Wound	No	12	No	Yes	No	No
10	25	M	ST239/t037	Blood	No	18	No	No	No	No
11	30	F	ST239/t037	Wound	No	10	No	Yes	No	No

### 3.3 Antimicrobial susceptibility profiles

All 11 MRSA isolates were resistant to cefoxitin and penicillin and confirmed as *mecA*-positive.

**Uniform resistance across both clones:** Clindamycin: 11/11 (100%) (including constitutive and inducible resistance confirmed by D-test), Gentamicin: 11/11 (100%), Vancomycin: 0/11 (0%) (MIC range: 0.5–2 μg/mL), Linezolid: 0/11 (0%).

**D-test results:** Of 11 isolates, 4 (36.4%) showed a positive D-test (inducible clindamycin resistance): 2/6 ST398 (33.3%) and 2/5 ST239 (40.0%). The remaining 7 showed constitutive resistance.

Clone-specific resistance:

**ST398/t011 (n=6):** Tetracycline: 6/6 (100%), Trimethoprim/sulfamethoxazole: 6/6 (100%), Erythromycin: 0/6 (0%), Levofloxacin: 0/6 (0%), Mupirocin: 0/6 (0%), Rifampicin: 0/6 (0%).

**ST239/t037 (n=5):** Erythromycin: 5/5 (100%), Levofloxacin: 5/5 (100%), Mupirocin: 5/5 (100%), Rifampicin: 4/5 (80%), Tetracycline: 0/5 (0%), Trimethoprim/sulfamethoxazole: 0/5 (0%).

These dichotomous, mutually exclusive resistance profiles enabled reliable preliminary clone discrimination prior to molecular confirmation.

### 3.4 Molecular typing results

#### 3.4.1 *SCCmec* typing

All ST398 isolates (n = 6) harbored SCC*mec* type V; all ST239 isolates (n = 5) harbored SCC*mec* type III. No other SCC*mec* types (I, II, IV) were detected.

#### 3.4.2 *agr* typing

All 11 MRSA isolates belonged to *agr* group I. No groups II, III, or IV were identified.

#### 3.4.3 *spa* typing

All ST398 isolates were assigned *spa* type t011 (repeat succession: 08-16-02-25-34-17-34); all ST239 isolates were assigned *spa* type t037 (15-12-16-02-25-17-24). No heterogeneity was observed within either clonal group.

#### 3.4.4 Multilocus sequence typing (MLST)

MLST confirmed six isolates as ST398 and five as ST239. No other sequence types were identified.

### 3.5 Virulence gene carriage

Significant differences in toxin gene carriage were observed:

**Panton-Valentine leukocidin (PVL):** Detected in 1/6 (16.7%) ST398/t011 isolates (Isolate No. 3, a 45-year-old male veterinarian with a purulent hand wound; PVL sequence deposited in NCBI GenBank under accession number PZ717229); absent in all ST239/t037 isolates.

**Toxic shock syndrome toxin (*tst*):** Detected in all 5 (100%) ST239/t037 isolates; absent in all ST398/t011 isolates.

**Enterotoxin C (*sec*):** Detected in all 5 (100%) ST239/t037 isolates; absent in all ST398/t011 isolates.

**Alpha-hemolysin (*hla*):** Detected ubiquitously in all 11 (100%) isolates.

Detailed resistance and virulence profiles are presented in Table 2.

**Table 2:** Antimicrobial resistance and virulence gene profiles of MRSA isolates.

Isolate	Clone	Resistance Pattern	PVL	tst	sec	hla
1	ST398/t011	PEN, FOX, CLI, GEN, TET, COT	–	–	–	+
2	ST398/t011	PEN, FOX, CLI, GEN, TET, COT	–	–	–	+
3	ST398/t011	PEN, FOX, CLI, GEN, TET, COT	+	–	–	+
4	ST398/t011	PEN, FOX, CLI, GEN, TET, COT	–	–	–	+
5	ST398/t011	PEN, FOX, CLI, GEN, TET, COT	–	–	–	+
6	ST398/t011	PEN, FOX, CLI, GEN, TET, COT	–	–	–	+
7	ST239/t037	PEN, FOX, CLI, GEN, ERY, LEV, MUP, RIF	–	+	+	+
8	ST239/t037	PEN, FOX, CLI, GEN, ERY, LEV, MUP, RIF	–	+	+	+
9	ST239/t037	PEN, FOX, CLI, GEN, ERY, LEV, MUP	–	+	+	+
10	ST239/t037	PEN, FOX, CLI, GEN, ERY, LEV, MUP, RIF	–	+	+	+
11	ST239/t037	PEN, FOX, CLI, GEN, ERY, LEV, MUP, RIF	–	+	+	+

PEN = penicillin; FOX = cefoxitin; CLI = clindamycin; GEN = gentamicin; TET = tetracycline; COT = trimethoprim/sulfamethoxazole; ERY = erythromycin; LEV = levofloxacin; MUP = mupirocin; RIF = rifampicin; PVL = Panton-Valentine leukocidin; tst = toxic shock syndrome toxin; sec = staphylococcal enterotoxin C; hla = alpha-hemolysin.

### 3.6 Comparison with first-half 2025 data

To assess continued detection, we compared our findings (July-December 2025, n = 11) with our previously published data (January-June 2025, n = 13) [23].

Key comparative findings.

**Table:** 

Parameter	First Half 2025 (Jan–Jun)	Second Half 2025 (Jul–Dec)	Total (Full Year)
Total MRSA isolates	13	11	24
ST398/t011 (LA-MRSA)	7 (53.8%)	6 (54.5%)	12 (50.0%)
ST239/t037 (HA-MRSA)	6 (46.2%)	5 (45.5%)	12 (50.0%)
SCC*mec* V (ST398)	7	6	13
SCC*mec* III (ST239)	6	5	11
*agr* type I (all)	13 (100%)	11 (100%)	24 (100%)
PVL-positive ST398	1 (14.3%)	1 (16.7%)	2 (16.7%)
*tst*-positive ST239	6 (100%)	5 (100%)	11 (100%)
*sec*-positive ST239	6 (100%)	5 (100%)	11 (100%)
Tetracycline + COT resistance (ST398)	7 (100%)	6 (100%)	12 (100%)
Mupirocin resistance (ST239)	6 (100%)	5 (100%)	11 (100%)
Rifampicin resistance (ST239)	5/6 (83.3%)	4/5 (80.0%)	9/11 (81.8%)

These data demonstrate: (1) similar MRSA prevalence across both periods; (2) similar distribution of ST398 and ST239 clones over 12 months; (3) conservation of clone-specific resistance phenotypes; (4) recurrent detection of PVL-positive ST398 in both halves; and (5) persistence of superantigen toxin genes (*tst*, *sec*) in all ST239 isolates.

A comprehensive comparative summary is presented in Table 3.

**Table 3:** Comparison of MRSA characteristics between first and second half of 2025.

Characteristic	First Half 2025 (Jan–Jun)	Second Half 2025 (Jul–Dec)
Total MRSA isolates	13	11
ST398/t011 (LA-MRSA)	7 (53.8%)	6 (54.5%)
ST239/t037 (HA-MRSA)	6 (46.2%)	5 (45.5%)
SCC*mec* V (ST398)	7	6
SCC*mec* III (ST239)	6	5
*agr* type I (all isolates)	13 (100%)	11 (100%)
PVL-positive ST398	1 (14.3%)	1 (16.7%)
*tst*-positive ST239	6 (100%)	5 (100%)
*sec*-positive ST239	6 (100%)	5 (100%)
Tetracycline + COT resistance (ST398)	7 (100%)	6 (100%)
Mupirocin resistance (ST239)	6 (100%)	5 (100%)
Rifampicin resistance (ST239)	5/6 (83.3%)	4/5 (80.0%)

## 4 Discussion

This prospective surveillance study provides evidence for continued detection of LA-MRSA ST398/t011 in a referral hospital in Bojnurd, northeastern Iran, co-circulating with the endemic HA-MRSA ST239/t037 clone. While ST398/t011 has been consistently detected over 12 months, our study design cannot confirm endemic establishment or human-to-human transmission.

### 4.1 Continued detection of LA-MRSA ST398

The repeated isolation of ST398/t011 from six independent patients over six months, with identical *spa*, *SCCmec*, *agr*, and resistance profiles, suggests either repeated introduction from a common agricultural reservoir or establishment of an ongoing transmission cycle involving livestock, farm workers, and their household contacts [33]. ST398 is the predominant LA-MRSA lineage worldwide, first described in France in 2003 and subsequently reported across Europe, North America, Asia, and Australia (8, 9). In Europe, prevalence is highest in the Netherlands, Denmark, and Germany, where intensive swine farming creates high-volume occupational exposure [34]. In Asia, ST398 has been reported in China, South Korea, and Malaysia, primarily in livestock and farm workers, with increasing clinical detection [35].

In Iran, livestock farming is a major economic sector, with approximately 8 million cattle, 50 million sheep, and 250 million poultry [36]. Despite this, clinical reports of LA-MRSA ST398 have been conspicuously absent. Our serial reports from 2025 fill this critical gap and suggest that ST398 may have been circulating undetected in Iranian agricultural communities. The 83.3% livestock exposure rate among ST398-infected patients provides an epidemiological association with agricultural reservoirs, consistent with international literature where occupational livestock contact is the strongest risk factor for ST398 colonization and infection [37,38].

### 4.2 Antimicrobial resistance patterns

All ST398/t011 isolates exhibited resistance to tetracycline, a hallmark of LA-MRSA lineages [39]. While we did not perform genotypic testing for specific tetracycline resistance genes, the observed phenotype is consistent with *tet(M)* or *tet(K)* genes on mobile genetic elements, reflecting selective pressure from tetracycline use in livestock production [40]. The European Union banned tetracycline for growth promotion in 2006, but it remains widely used in Asia, including Iran [41]. The consistent co-resistance to trimethoprim/sulfamethoxazole is also characteristic of LA-MRSA and likely reflects co-selection on mobile elements [42].

### 4.3 PVL-positive ST398: A signal warranting vigilance

The detection of *PVL* in one ST398/t011 isolate (16.7%) in this period, coupled with a *PVL*-positive ST398 in our first-half report [23], is notable. *PVL* is a bicomponent pore-forming toxin causing leukocyte destruction and tissue necrosis, strongly associated with severe necrotizing infections [14,43]. Classical LA-MRSA ST398 strains were historically *PVL*-negative [44]. However, recent reports from Europe, Asia, and Australia have documented *PVL*-positive ST398, indicating horizontal acquisition of *PVL*-encoding prophages [16,17,45]. This may represent a step toward enhanced human adaptation and transmissibility.

However, we caution against overinterpretation. Only a single PVL-positive isolate was identified, and the patient had no evidence of severe disease (no ICU admission, no surgery, discharged after 7 days of vancomycin). Our findings are hypothesis-generating, not definitive evidence of increased virulence. The detection of PVL-positive ST398 in two independent patients over two consecutive periods warrant continued surveillance.

### 4.4 HA-MRSA ST239: an endemic healthcare threat

The continued detection of ST239/t037/SCCmec III/agr I in 5 patients, with identical multidrug resistance profiles to first-half isolates, confirms the deep entrenchment of this pandemic hospital-associated clone in Iranian healthcare settings [18,19]. ST239 (CC8) is one of the most successful healthcare-associated MRSA clones globally, documented in South America, Europe, Asia, and Australia [46,47]. It is characterized by SCC*mec* type III (carrying multiple resistance genes), multidrug resistance (β-lactams, macrolides, aminoglycosides, fluoroquinolones), and frequent carriage of superantigen toxins (*tst*, *sec*).

All ST239 isolates carried both *tst* and *sec*. *TSST*-1 is a superantigen triggering massive T-cell proliferation and cytokine release, leading to toxic shock syndrome [48]. *SEC* is an emetic toxin associated with food poisoning and may contribute to immune evasion [49]. The universal carriage of these genes suggests they are stable components of Iranian ST239 strains.

The 80–83% rifampicin resistance and 100% mupirocin resistance among ST239 isolates are concerning. Mupirocin is widely used for MRSA decolonization; resistance is associated with decolonization failure and nosocomial transmission [50]. Rifampicin is a key component of combination therapy for severe MRSA infections (osteomyelitis, prosthetic joint infections); resistance significantly limits therapeutic options [51]. The continued detection of these resistances over 12 months suggests ongoing selective pressure.

### 4.5 Clinical implications of Co-circulation

The co-circulation of two genetically and epidemiologically distinct MRSA lineages, one community/livestock-associated (ST398) and one healthcare-associated (ST239), within a single hospital presents unique challenges:

**Diagnostic:** Different resistance profiles require different empirical antibiotic selection; PVL testing may be warranted for severe ST398 infections.

**Therapeutic:** ST398 remains susceptible to erythromycin, levofloxacin, mupirocin, and rifampicin, agents to which ST239 is resistant. ST239 remains susceptible to tetracycline and COT, agents to which ST398 is resistant. Both remain susceptible to vancomycin and linezolid.

**Infection control:** ST239 transmission is primarily nosocomial; ST398 transmission is primarily zoonotic/community-acquired, with potential secondary nosocomial spread. Different reservoirs require different intervention strategies.

The identical *spa* and MLST types observed over 12 months suggest either repeated introduction from a common agricultural reservoir or establishment of an in-hospital reservoir. Distinguishing between these scenarios requires whole-genome sequencing (WGS) and integrated human-animal-environmental sampling [52].

### 4.6 Regional and global context

In the Middle East, LA-MRSA ST398 data remain extremely limited. Sporadic reports describe ST398 in livestock in Turkey, Iraq, and Saudi Arabia, but clinical isolation from human infections is exceptionally rare [53,54]**.** To our knowledge, this is the first documentation of continued clinical detection of LA-MRSA ST398 in Iran, although this should be interpreted with caution given limitations in national surveillance. The absence of prior reports may reflect under-surveillance rather than true absence. Our serial reports position Iran as a sentinel site for emerging LA-MRSA in the region.

In Asia, ST398 prevalence varies widely. In China, high prevalence in swine and farm workers with increasing clinical reports, including *PVL*-positive strains [35]. In South Korea, established LA-MRSA in swine with sporadic human infections [55]. In Malaysia/Thailand, emerging detection in livestock and humans [56]. In India, predominance of ST772 and ST22; ST398 rare [57].

The acquisition of *PVL* by ST398 mirrors global trends. Coombs et al. [16] reported *PVL*-positive ST398 in Australia associated with severe community-onset infections. Wu et al. [45] documented *PVL*-positive ST398 in China with enhanced virulence in murine models. Our findings add Iran to the growing list of countries where LA-MRSA ST398 may be undergoing genetic changes suggestive of increased human adaptation.

### 4.7 Limitations

Several limitations should be acknowledged. First, the small sample size (n = 11) limits statistical power; confidence intervals were wide, and p-values should be interpreted descriptively. Second, the single-center design limits generalizability. Third, the absence of WGS is a major limitation, we cannot assess genetic relatedness, distinguish human-to-human transmission from repeated introductions, or identify mutations associated with virulence or resistance evolution. Fourth, we did not perform animal or environmental sampling to confirm livestock reservoirs. Fifth, the lack of a control group (MRSA-negative or MSSA patients) prevents inference that livestock exposure is a specific risk factor for ST398 acquisition. Sixth, detailed follow-up was limited. Seventh, we were unable to test for human immune evasion genes (chp, scn, sak) that would distinguish human-adapted versus livestock-adapted ST398 lineages.

## 5 Conclusion

This six-month surveillance study documents continued detection of LA-MRSA ST398/t011 at a referral hospital in northeastern Iran, co-circulating with endemic HA-MRSA ST239/t037 over a 12-month period. The detection of a PVL-positive ST398 isolate, while based on a single case with mild clinical presentation, warrants continued vigilance and further investigation. These findings are descriptive and hypothesis-generating, not confirmatory, given the small sample size and single-center design. Our results underscore the need for integrated One Health surveillance, including WGS and animal-environmental sampling, to better understand the transmission dynamics and clinical significance of LA-MRSA ST398 in Iran and the broader Middle East region.

## CRediT authorship contribution statement

**Seyed Ahmad Hashemi:** Writing – original draft, Methodology, Data curation. **Ali Haghbin:** Writing – original draft, Methodology, Conceptualization. **Ahmad Vosughi Motlagh:** Methodology, Data curation. **Amir Azimian:** Writing – review & editing, Writing – original draft, Visualization, Validation, Supervision, Software, Resources, Project administration, Methodology, Investigation, Funding acquisition, Formal analysis, Data curation, Conceptualization.

## Ethical approval

Approved by the Ethics Committee of North Khorasan University of Medical Sciences (IR.NKUMS.REC.1401.012).

## Consent for publication

Not applicable.

## Data availability statement

The sequence data generated in this study (spa repeat sequences and MLST allele sequences) have been deposited in NCBI GenBank under accession numbers: spa sequences: PZ717212-PZ717213; MLST allele sequences: PZ717214-PP987649. The PVL gene sequence from the PVL-positive isolate has been deposited under accession number PZ717229. Raw data and other supporting information are available from the corresponding author upon reasonable request.

## Sequence accession numbers

The sequence data (spa repeat sequences and MLST allele sequences) have been deposited in NCBI GenBank under accession numbers: spa sequences: PZ717212-PZ717213; MLST allele sequences: PZ717214-PP987649. The PVL gene sequence from the PVL-positive isolate has been deposited under accession number PZ717229.

## Generative AI statement

During the preparation of this work, the authors used Grammarly for language refinement and grammar checking. After using these tools, the authors reviewed and edited the content as needed and take full responsibility for the content of the published article.

## Funding

This work was supported by the North Khorasan University of Medical Sciences [Grant Number: 980345].

## Declaration of competing interest

The authors declare that they have no known competing financial interests or personal relationships that could have appeared to influence the work reported in this paper.
